# Validity and reliability of the Spanish version of the “Wijma Delivery Expectancy/Experience Questionnaire” (W-DEQ-B)

**DOI:** 10.1371/journal.pone.0249942

**Published:** 2021-04-26

**Authors:** Juan Roldán-Merino, Celia Maria Ortega-Cejas, Teresa Lluch-Canut, Mariona Farres-Tarafa, Ainoa Biurrun-Garrido, Irma Casas, Mª Isabel Castrillo-Pérez, Mª Mercedes Vicente-Hernández, Marta Jimenez-Barragan, Raquel Martínez-Mondejar, Barbara Hurtado-Pardos, Sandra Cabrera-Jaime

**Affiliations:** 1 Campus Docent, Sant Joan de Déu—Fundació Privada, School of Nursing, University of Barcelona, Barcelona, Spain; 2 Research Group GIES (Grupo de investigación en Enfermería, Educación y Sociedad), Barcelona, Spain; 3 Research Group GEIMAC (Consolidated Group 2017–1681: Group of Studies of Invariance of the Instruments of Measurement and Analysis of Change in the Social and Health Areas), Barcelona, Spain; 4 Midwife, Sexual and Reproductive Health Clinic (ASSIR), Mollet del Vallès Barcelona, Barcelona, Spain; 5 Faculty of Medicine and Health Sciences, University of Barcelona, Barcelona, Spain; 6 Member Research Group GRISIMula (Grupo emergente 2017 SGR 531; Grupo en Recerca Enfermera en Simulación), Barcelona, Spain; 7 Secretary, GRISCA Research Group (Nursing Simulation in Catalonia and Andorra Research Group), Barcelona, Spain; 8 Universitat Autònoma de Barcelona, Barcelona, Spain; 9 Preventive Medicine Service, Hospital Germans Trias i Pujol, Barcelona, Spain; 10 Research Group Innovation in Respiratory Infections and Tuberculosis Diagnosis (Group Consolidat 2017 SGR 494), Barcelona, Spain; 11 Midwife, Sexual and Reproductive Health Clinic (ASSIR) La Riera, Badalona, Barcelona, Spain; 12 Midwife, Sexual and Reproductive Health Clinic (ASSIR) Doctor Barraquer (Sant Adrià del Besòs), Barcelona, Spain; 13 Midwifery Coordinator, Sexual and Reproductive Health Clinic (ASSIR) Fundació Assistencial Mútua Terrassa, (Terrassa) Midwife, Sexual and Reproductive Health Clinic (ASSIR) Rambla Terrassa, (Terrassa) Docente en Fundació Universitaria del Bages, Barcelona, Spain; 14 Corporate Care Management, Institut Català d’Oncologia (ICO), L’Hospitalet de LLobregat, Barcelona, Spain; 15 GRIN Group, IDIBELL, Institute of Biomedical Research, Barcelona, Spain; Murcia University, Spain, SPAIN

## Abstract

The Wijma Delivery Expectancy/Experience Questionnaire (W-DEQ-B) is an instrument that allows the experiences around fear of childbirth to be examined after the birth. It is currently the most widely used to measure different aspects related to the fear of childbirth and enables healthcare and additional assistance to women after birth to be adapted according to their needs. The objective of this study was to translate the W-DEQ-B into Spanish and analyse its reliability and validity. The study was carried out in two phases: (1) transcultural adaption of the questionnaire to Spanish and (2) a transversal study in a sample of 190 postpartum women from Sexual and Reproductive Health Clinics in the province of Barcelona (Spain). The psychometric properties were examined in terms of reliability (internal consistency and temporal stability) and construct validity (confirmatory factorial analysis [CFA] and exploratory factorial analysis [EFA]). The results of the CFA did not confirm unidimensionality of the W-DEQ-B questionnaire. The EFA suggested four very similar, but not identical, dimensions to those obtained in other studies in which the W-DEQ-B has been evaluated. Both the Cronbach’s alpha and the omega coefficient were adequate for the total questionnaire and for each of the four dimensions. The results of this study confirm that the W-DEQ-B is multi-dimensional. In the Spanish version of the W-DEQ-B-Sp four dimensions have been identified that allow the experiences around fear of childbirth to be examined after the birth. The Spanish version of the WDEQ-B (WDEQ-B-Sp) is reliable and valid for the measurement of fear of childbirth in clinical practice and for use in future research.

## Introduction

Fear of childbirth has aroused an increased interest in the scientific community in recent years [1]. The effects of fear on the development of the pregnancy and the birth have been widely studied [1]. The prevalence of fear of childbirth is difficult to establish according to the different research studies and populations in which it has been evaluated, however a recent meta-analysis evaluated and documented global prevalence of 14% [2]. Although it has been estimated that fear of childbirth is more prevalent amongst nulliparous women [2,3] it has also been seen in multiparous women in relation to a negative birth experience [4–7] and/or a previous emergency cesarian section [8].

Bearing in mind that the birth is not only a physiological event, but also implies a psychological process of transition into motherhood [9], the situation undergone by a woman during labor can determine her attitude and life experience regarding it [10]. In fact, women describe the fear as “a ghost that gets into my head” or a “huge knot in my stomach” and “remembering waking up terrified in a cold sweat because of thoughts of the coming birth,” which leads them to avoid it, as one strategy amongst others, to cope with it [11].

The acquisition of fear before and after birth could also be related to each other according to several authors, so fear of childbirth during pregnancy could be related to fear during labor and fear after the birth [7,12]. In fact, some studies mention that women during pregnancy try to “keep away from detailed information about birth,” or “avoid prenatal classes” [11]. In turn, the studies show that women get to a point of feeling guilty for “feeling like this”, or “feeling that you are not as good as other people” because you feel frightened” [11].

On the other hand, expectations generated around the birth can be a poor fit with the experience lived during birth [13]. This is observed in cases of pregnant women who want a vaginal birth, but for various reasons finally have to have an emergency cesarian. In these cases the women present a higher risk of developing serious post-birth fear [12], therefore it can be said that the experience of birth can become traumatic [8].

Furthermore, it has also been observed that pregnant women with fear of childbirth, who requested an elective cesarean section but who finally have a vaginal delivery, are more likely to suffer from post-traumatic stress syndrome (PTSD) [14].

Several studies have analyzed the relation between fear of birth and a higher probability of postpartum depression and the development of post-traumatic stress disorder [15,16] and consider the fear to be a risk factor for developing the latter of these [17,18], because negative experiences associated with fear can become "imprinted" in women, promoting the onset of posttraumatic stress syndrome [19]. In addition, fear of postpartum childbirth has been associated with difficulties in establishing maternal-filial bonding [20] as well as a perception of increased negative affection for their infant [21].

The Wijma Delivery Expectancy/Experience Questionnaire (W-DEQ) was developed by Wijma *et al*, at the end of the 1990s. The questionnaire consists of two versions that evaluate fear of childbirth through expectations (Version A) and the experience lived during labor (Version B). Both versions have been translated and validated in multiple languages and for this reason it is the most commonly used instrument used in clinical practice to evaluate fear of childbirth [22]. Specifically, the Version B of the questionnaire has been translated and validated in Italian, Hindu, Turkish, German, Farsi and Japanese [23–28].

Although perinatal mental health arouses interest, and despite the implementation of screening for mental disorders for pregnant and postpartum women in Spain, there are no validated instruments that permit the identification of pregnant women with fear of childbirth early on in the postpartum period. Although the validation of version A of this questionnaire in Spanish has recently been published [29] with the aim of having an instrument to assess fear of childbirth during pregnancy, the existence of a tool of this type for the postpartum period would make it possible to detect the psychological needs of women after childbirth, and to implement interventions to improve the recall of the childbirth experience.

For this reason, the objective of this study was to translate the Wijma Delivery Expectancy/Experience Questionnaire (W-DEQ-B) into Spanish and analyse its validity and reliability.

## Methods

### Design

The study was carried out in two phases. The first consisted of adapting the W-DEQ-B to Spanish; and in the second phase the metrics of the Spanish version were analyzed.

### Participants and setting

In this study 190 postpartum women from various Centers of Sexual and Reproductive Health participated. Patients were recruited during the last pregnancy follow-up visits. They were provided with written information about the study and an informed consent form that they had to sign if they agreed to voluntarily participate in the research. The questionnaires were administered during the first 24 hours after delivery during the admission of the puerperal women at the respective referral hospitals. The inclusion criteria were to be over 18 years of age and without difficulties to read and complete the questionnaire in Spanish. Women who had a history of perinatal death were excluded. The women were recruited consecutively during the period of the study from January to December of 2019.

To estimate the sample size the recommendations of various authors who consider that between 5 and 20 participants should be included for each item that makes up the questionnaire were followed [30,31]. In this study it was agreed to include a minimum of 5 postpartum women for each item that makes up the questionnaire.

### Variables and source of information

The W-DEQ-B consists of 33 items. In the original version all the items are grouped in one single dimension. Each item is evaluated using an ordinal scale of 0 to 5. The extremes of the replies (0 and 5 respectively) correspond with the opposites of a feeling or a thought. The minimum score is 0 and the maximum 165. The scores for the items 2, 3, 6, 7, 8, 11, 12, 15, 19, 20, 24, 25, 27 and 31 must be reversed.

The original Wijma study [32] a Cronbach alpha of 0.87 was obtained. All the items of the W-DEQ-B questionnaire were included as variables.

Other variable were also included such as: age, employment status, level of studies, number of births, presence or not of a partner, the type of birth and whether there were perinatal complications.

### Procedure

The process of cultural adaption of the Version B of the W-DEQ questionnaire was performed following the Standards for Educational and Psychological Testing [33].

The English version of the questionnaire was translated to Spanish by 2 sworn translators whose mother-tongue was Spanish and who were competent in English to obtain two versions of the W-DEQ-B in Spanish. These were evaluated by a committee of experts that included 3 midwives, a gynecologist, a nurse specialized in validating instruments and a psychologist with experience in the area of sexual and reproductive health.

The consensus version was translated to English by two new sworn translators, whose mother-tongue was English and who were competent in Spanish. Subsequently, the committee of experts compared the two versions with the original the questionnaire and did not find any discrepancies that needed modification.

### Pilot study

A pilot study was carried out with a total of 25 postpartum women to evaluate the comprehension and clarity of the items as well as the format and time required to complete the questionnaire. The estimated average time oscillated between 10 and 15 minutes and the participants confirmed that it was easy to complete. After the debriefing it was not necessary to make any changes in the content or the format. The Spanish version was named W-DEQ-B-Sp.

### Statistical analysis

First a confirmatory factorial analysis (CFA) was carried out to test the unidimensional model of the original scale proposed by Wijma [32] and secondly an exploratory factorial analysis was performed to determine the number of possible factors in the Spanish version. Given the ordinal nature of the items, the generalized least squares method was used for the CFA to estimate the parameters [34]. The adjustment of the model was determined from the following: the chi-squared goodness-of-fit test, the ratio between chi-squared and the degrees of freedom (χ2/df), the BBNFI (Bentler Bonnet Normed Fit Index), the BBNNFI (Bentler Bonnet Non-Normed Fit Index), the CFI (comparative fit index), the GFI (goodness-of-fit index), the AGFI (adjusted goodness-of-fit index), and RMSEA (Root Mean Standard Error Approximation). The criteria for a good fit were BBNFI, BBNNFI, CFI, GFI and AGFI values above 0.90 [35–37], and RMSEA values were to be below 0,08 [34,38]. The reduced Chi-squared, defined as the ratio between the Chi-squared value and the number of degrees of freedom; values between 2 and 6 were considered acceptable [39].

In order to study the factorial validity of the questionnaire W-DEQ-B-Sp in the Spanish sample, an EFA was performed, previously examining its pertinence with the Kaiser, Meyer, Olkin Measure of Sampling Adequacy (KMO) and the Bartlett’s test of sphericity, using the classical implementation of Horn’s Parallel Analysis to extract the dimensions [40]. This method allows the adequate identification of the number of dimensions of the questionnaire [41]. The EFA was adjusted to the polychoric correlation matrix given the nature of the items [42].

The function adjustment selected was that of robust unweighted least squares with adjustment statistics mean variance corrected [43]. The factors were rotated using the Robust Promin rotation [44].

In the same way, the indexes for Cronbach’s alpha confidence and omega were calculated for each resulting factor and for the total questionnaire. The values considered adequate were Cronbach’s alpha above 0.70 [45] and an omega index above 0.80 [46]. The temporal stability, or test-retest was analyzed using the intraclass correlation coefficient in the sample of 150 postpartum women. The temporal stability was analyzed after 15 days.

The Sample characteristics were explained using descriptive statistics using frequencies and percentages, measures of central tendency and dispersion.

The EFA was performed with the Factor Analysis [43] and CFA models were estimated using structural equation modeling (EQS 6.4 for Windows, Multivariate Software, Inc., Encino, CA, USA).

### Ethical considerations

The study has been carried out with the consent of the original author of the questionnaire. The study was approved by the Clinical Research Ethics Committee of the Jordi Gol Foundation (code P14/106) and the Human Research Ethics Committee at the Germans Trias i Pujol Hospital (code PI14-074). All women were informed of the purpose of the study at the last pregnancy follow-up visits. They were given an information sheet on the purpose of the study and an informed consent form to be signed if they agreed to voluntarily participate in the study.

## Results

### Characteristics of participants

The characteristics of the participants are shown in Table 1. A total of 190 postpartum women were included in the study. The average age was 33.1 (SD 5.0) and 34% were multiparous. 89.5% said that they were in work and 78.9% had university level studies.

**Table 1:** Sociodemographic and clinical characteristics of the study sample.

Characteristics	n	%
Age (mean, SD)	33,0 (5,0)	
Partner		
With partner	185	97,4
Without partner	5	2,6
Level of studies		
Primary	8	4,2
Secondary	32	16,8
University	150	78,9
Employment status		
Working	170	89,5
Out of work	20	10,5
Parity		
Nulliparous	129	65,8
Multiparous	65	34,2
Type of birth		
Eutocic	106	55,8
Vacuum	24	12,6
Forceps	10	5,3
Thierry’s spatulas		2,1
Cesarean	46	24,2
Complication		
Yes	22	11,6
No	168	88,4

### Construct validity

#### Confirmatory Factor Analysis (CFA)

To verify the unidimensionality of the original version of the questionnaire an CFA was performed. The model showed a poor fit (for example BBMFI = 0.53, BBNI = 0.59, CFI = 0.61 and a RMSEA of 0.11) (Table 2).

**Table 2:** Goodness-of-fit indexes for the confirmatory model W-DEQ-B-Sp.

INDEX	VALUE
BBNFI	0.538
BBNFI	0.591
CFI	0.617
GFI	0.937
AGFI	0.928
RMSEA	0.114
Goodness of fit test	χ^2^ = 1707,780; *gl* = 495; *p* < 0.0001
Reason for fit	χ^2^/*gl* = 3,45

BBNFI, Bentler Bonnet Normed Fit Index; BBNNFI, Bentler Bonnet Non-Normed Fit Index; CFI: Comparative Fit Index; GFI, Goodness of Fit Index; AGFI, Adjusted Goodness of Fit Index; RMSEA, root mean standard error of approximation; df, degrees of freedom.

### Exploratory factor analysis (EFA)

An exploratory factorial analysis was performed on the Spanish sample to check whether multiple dimensions existed for the W-DEQ-B-Sp in the same way as had been done for the different languages for which the W-DEQ-B questionnaire had previously been validated. The value of the KMO was 0.91, and Bartlett’s test of sphericity significance level was χ2 = 2039.7; df = 528; p = 0.00001. Using parallel analysis 4 dimensions were identified. The percentage of variance explained by the 4 dimensions is 59.3%. The indexes for the goodness of fit for the 4-dimension model are shown in Table 3.

**Table 3:** Indexes of goodness of fit of the exploratory factor analysis of the model for four dimensions the W-DEQ-B-Sp.

INDEX	VALUE	95% confidence interval
CFI	0.996	0.990–1.016
GFI	0.980	0.976–0.986
AGFI	0.974	0.968–0.982
RMSEA	0.026	0.003–0.042
Goodness of fit test	χ^2^ = 454,014; *gl* = 402; *p* < 0.0372	
Reason for fit	χ^2^/*gl* = 1,12	

**CFI:** Comparative Fit Index. **GFI**: Goodness of Fit Index. **AGFI**: Adjusted Goodness of Fit Index. **RMSEA**: Root Mean Standard Error of Approximation.

The four factors have been defined as ‘Concerns about childbirth and child’, ‘Isolation’, ‘Fear’ and ‘Lack of self-efficacy’.

The variables that configured each factor and the percentage of variation explained for each of them are shown in Table 4.

**Table 4:** Loading matrix related to the exploratory factor analysis solution.

Item No.	Description	Factor 1 Concerns about childbirth and child	Factor 2 Isolation	Factor 3 Fear	Factor 4 Lack of self-efficacy
1	Fantastic			.449	
2	Frightful			.601	
3	Lonely		.734		
4	Strong				.485
5	Confident				.609
6	Afraid			.437	
7	Deserted		.884		
8	Weak			.595	
9	Safe			.498	
10	Independent				.424
11	Desolate			.	.334
12	Tense			557	
13	Glad				.764
14	Proud				.859
15	Abandoned		.738		
16	Composed				.569
17	Relaxed			.546	
18	Happy				.667
19	Panic			.609	
20	Hopelessness			.522	
21	Longing for the child				.658
22	Self-confidence				.822
23	Trust				.736
24	Pain			.686	
25	I will behave extremely badly			.672	
26	I allowed my body to take total control	.			.424
27	I lost totally control of myself			.596	
28	Enjoyable	.620			
29	Natural	.769			
30	Should be	.759			
31	Dangerous	.770			
32	Fantasies that your child die during labour/delivery	.541			
33	Fantasies that your child will be injured during labour/delivery	.628			
Percent of variance		**38,3**	**7,7**	**6,9**	**6,2**

### Internal consistency and temporal stability

The results of the W-DEQ-B-Sp related to reliability and test-retest temporal stability are shown in Table 5. A Cronbach’s alpha of 0.93 was obtained for the complete questionnaire and the values for all the dimensions oscillated between 0.79 and 0.88. The omega coefficient (ω) for the complete questionnaire and for each of the factors was above 0.85. ICC analysis demonstrated that the test–retest reliability was 0.93 (95% confidence interval 0.91–0.53) and this value was greater than 0.78 for the 4 dimensions.

**Table 5:** W-DEQ-B-Sp Cronbach’s alpha coefficient, omega coefficient and ICC test-retest (n = 155).

Factor	Cronbach’s alpha	Omega (ω)	ICC (CI 95%)
**F.1.** Concerns about childbirth and child	.820	.858	.912 (.879-.936)
**F.2.** Isolation	.779	.889	.792 (.714-.848)
**F.3.** Fear	.884	.902	.907 (.873-.933)
**F.4.** Lack of self-efficacy	.876	.919	.781 (.708-.835)
**Total**	.932	.946	.936 (.912-.953)

**ICC:** Intraclass correlation coefficient; **CI**: Confidence interval.

## Discussion

The objective of this study was to adapt the Wijma Delivery Expectancy/Experience Questionnaire (W-DEQ-B) to Spanish and analyze the psychometric properties. It is a questionnaire consisting of 33 items grouped in one single dimension. The W-DEQ-B was developed by Wijma and collaborators (1998) to examine the experiences around fear of childbirth after a birth [32]. It is an adequate instrument to measure feelings, emotions, experiences and fear a woman has of childbirth after having experienced it. The experiences regarding fear of childbirth signal what a woman thinks and feels about the birth. Thus the W-DEQ-B is an effective measurement instrument to permit the determination of attention and additional care that women might need after birth. Additionally, it enables the detection of psychological needs of women in the process of transitioning into motherhood.

Regarding the construct validity, the reason for performing a CFA in our study was to determine the unidimensionality on which the original questionnaire was designed by Wijma and collaborators [32]. The method used for the CFA was that of generalized least squares, which is suitable when the items on the questionnaire are of an ordinal nature. The majority of the indexes of the model analyzed presented a poor fit: BBNFI (Bentler Bonnet Normed Fit Index), BBNNFI, (Bentler Bonnet Non-Normed Fit Index), CFI (Comparative Fit Index), RMSEA (Root Mean Square Error of Approximation), and normalized Chi-squared. These results confirm that the questionnaire is not unidimensional and so it behaves like a multifactorial instrument. These findings are similar to other studies done to validate this version [23–28,47,48].

As a consequence of these results an EFA was performed to test what could be expected of the model in the Spanish population. The method used for the EFA was to use a classical implementation of Horn’s Parallel Analysis [40], this model allows the identification of the true number of dimensions of a questionnaire [40,41,49].

The analysis identified 4 dimensions similar, but not identical, to those identified in other studies to validate the WDEQ-B [47,48,50].

The reliability of the questionnaire has been analyzed using the internal consistency and temporal stability (test-retest). The internal consistency was analyzed using Cronbach’s alpha and the omega coefficient. The Cronbach’s alpha for the complete questionnaire was 0.932 and the values for each of the dimensions was greater than 0.779. Values considered adequate are those between 0.70 and 0.90 [51,52] and values over 0.90 are considered excellent [53]. The values obtained in this study have been similar to those obtained in other studies [23–27,47,48,50].

The omega coefficient (w) also gave adequate values, 0.932 being obtained for the complete questionnaire and 0.859 for all the dimensions. Values over 0.80 are considered adequate [46].

In this study the temporal stability (test-retest) was also analyzed using the intraclass correlation coefficient. From 190 postpartum women participating at the start of the study only 150 completed the questionnaire a second time within 15 days. Values greater than 0.90 are considered to show excellent agreement and those between 0.71 and 0.90 are considered to show good agreement [54–56]. In this study the ICC was excellent for the total questionnaire and for all the dimensions, with the exception of the dimensions 2 and 4, which have obtained good agreement (0.792 and 0.781 respectively). No other study has analyzed the temporal stability, or test-retest.

### Limitations

This study has certain limitations, which should be borne in mind. Firstly, the women were selected consecutively and took part voluntarily in the study, so there may be bias in the selection. However, a large number of women from various centers in the province of Barcelona participated and the profile of these women may not be representative to the rest of the women in the Spanish population, so more research in other areas of Spain is needed. Secondly, as previous evaluation had not been made, the sensitivity to change could not be analyzed. In future longitudinal, or post-intervention studies this can be studied.

## Conclusions

The findings of the study confirm the results of other studies which have determined tha the WDEQ-B is multidimensional. 4 dimensions have been identified in the Spanish version of the questionnaire, the WDEQ-B-Sp, which allow the detection of the psychological needs of women during the process of transition into motherhood. It is a self-administered questionnaire, which requires little time to complete and has good psychometric properties in terms of reliability and construct validity. The statistical methods used in this study mean that it adds solid evidence to support the use of the questionnaire in the Spanish population.

Finally, having this questionnaire will allow the identification of patients with postpartum fear for the implementation of early interventions to reduce the possibility of developing other mental illnesses during the postpartum period. Future research on this subject is also needed.

## Supporting information

S1 File (XLS)

S2 File (DOC)

## References

[1] Dai L, Zhang N, Rong L, Ouyang YQ. Worldwide research on fear of childbirth: A bibliometric analysis. PLoS One. 2020. 10.1371/journal.pone.0236567 32726336 PMC7390386

[2] O’Connell MA, Leahy-Warren P, Khashan AS, Kenny LC, O’Neill SM. Worldwide prevalence of tocophobia in pregnant women: systematic review and meta-analysis. Acta Obstet Gynecol Scand. 2017;96(8):907–20. 10.1111/aogs.13138 28369672

[3] Rouhe H, Salmela-Aro K, Halmesmäki E, Saisto T. Fear of childbirth according to parity, gestational age, and obstetric history. BJOG An Int J Obstet Gynaecol. 2009;116(1):67–73. 10.1111/j.1471-0528.2008.02002.x 19055652

[4] Størksen HT, Garthus-Niegel S, Vangen S, Eberhard-Gran M. The impact of previous birth experiences on maternal fear of childbirth. Acta Obstet Gynecol Scand. 2013;92(3):318–24. 10.1111/aogs.12072 23278249

[5] Elvander C, Cnattingius S, Kjerulff KH. Birth experience in women with low, intermediate or high levels of fear: Findings from the first baby study. Birth. 2013;40(4):289–96. 10.1111/birt.12065 24344710 PMC3868996

[6] Dencker A, Nilsson C, Begley C, Jangsten E, Mollberg M, Patel H, et al. Causes and outcomes in studies of fear of childbirth: A systematic review. Women and Birth. 2019;32(2):99–111. 10.1016/j.wombi.2018.07.004 30115515

[7] Rondung E, Thomtén J, Sundin Ö. Psychological perspectives on fear of childbirth. J Anxiety Disord. 2016;44:80–91. 10.1016/j.janxdis.2016.10.007 27788373

[8] Nilsson C, Bondas T, Lundgren I. Previous Birth Experience in Women With Intense Fear of Childbirth. JOGNN—J Obstet Gynecol Neonatal Nurs. 2010;39(3):298–309. 10.1111/j.1552-6909.2010.01139.x 20576072

[9] Lukasse M, Schei B, Ryding E. Prevalence and associated factors of fear of childbirth in six European countries. Sex Reprod Healthc [Internet]. 2014 10 1 [cited 2020 Dec 28];5(3):99–106. Available from: https://pubmed.ncbi.nlm.nih.gov/25200969/. 10.1016/j.srhc.2014.06.007 25200969

[10] Reisz S, Jacobvitz D, George C. Birth and motherhood: Childbirth experience and mothers’ perceptions of themselves and their babies. Infant Ment Health J. 2015;36(2):167–78. 10.1002/imhj.21500 25704337

[11] Eriksson C, Jansson L, Hamberg K. Women’s experiences of intense fear related to childbirth investigated in a Swedish qualitative study. Midwifery. 2006;22(3):240–8. 10.1016/j.midw.2005.10.002 16603282

[12] Sluijs AM, Wijma K, Cleiren MPHD, JMM van Lith, Wijma B. Preferred and actual mode of delivery in relation to fear of childbirth. J Psychosom Obstet Gynecol. 2020;41(4):266–74. 10.1080/0167482X.2019.1708319 31896292

[13] Lundgren I. Swedish women’s experience of childbirth 2 years after birth. Midwifery. 2005;21(4):346–54. 10.1016/j.midw.2005.01.001 16024149

[14] Olieman RM, Siemonsma F, Bartens MA, Garthus-Niegel S, Scheele F, Honig A. The effect of an elective cesarean section on maternal request on peripartum anxiety and depression in women with childbirth fear: A systematic review. BMC Pregnancy Childbirth. 2017;17(1):195. 10.1186/s12884-017-1371-z 28629393 PMC5477251

[15] Söderquist J, Wijma B, Thorbert G, Wijma K. Risk factors in pregnancy for post-traumatic stress and depression after childbirth. BJOG An Int J Obstet Gynaecol. 2009;116(5):672–80. 10.1111/j.1471-0528.2008.02083.x 19220236

[16] Wijma K, Alehagen S, Wijma B. Development of the Delivery Fear Scale. J Psychosom Obstet Gynecol. 2002;23(2):97–107. 10.3109/01674820209042791 12189903

[17] Çapik A, Durmaz H. Fear of Childbirth, Postpartum Depression, and Birth-Related Variables as Predictors of Posttraumatic Stress Disorder After Childbirth. Worldviews Evidence-Based Nurs. 2018;15(6):455–63. 10.1111/wvn.12326 30281197

[18] Ayers S, Bond R, Bertullies S, Wijma K. The aetiology of post-traumatic stress following childbirth: A meta-analysis and theoretical framework. Psychol Med. 2016;46(6):1121–34. 10.1017/S0033291715002706 26878223

[19] Olza I, Uvnas-Moberg K, Ekström-Bergström A, Leahy-Warren P, Karlsdottir SI, Nieuwenhuijze M, et al. Birth as a neuro-psycho-social event: An integrative model of maternal experiences and their relation to neurohormonal events during childbirth. PLoS ONE. 2020. 10.1371/journal.pone.0230992 32722725 PMC7386571

[20] Challacombe FL, Nath S, Trevillion K, Pawlby S, Howard LM. Fear of childbirth during pregnancy: associations with observed mother-infant interactions and perceived bonding. Arch Womens Ment Health. 2020;17. 10.1007/s00737-020-01098-w 33336315 PMC8116271

[21] Vismara L, Sechi C, Neri M, Paoletti A, Lucarelli L. Maternal perinatal depression, anxiety, fear of birth, and perception of infants’ negative affectivity at three months. J Reprod Infant Psychol. 2020;11:1–12. 10.1080/02646838.2020.1843612 33172285

[22] Richens Y, Lavender DT, Smith DM. Fear of birth in clinical practice: A structured review of current measurement tools. Sex Reprod Healthc. 2018;16:98–112. 10.1016/j.srhc.2018.02.010 29804785

[23] Fenaroli V, Saita E. Fear of childbirth: A contribution to the validation of the Italian version of the Wijma Delivery Expectancy/Experience Questionnaire (WDEQ). TPM—Testing, Psychom Methodol Appl Psychol. 2013;20(2):131–54.

[24] Jha P, Larsson M, Christensson K, Svanberg AS. Fear of childbirth and depressive symptoms among postnatal women: A cross-sectional survey from Chhattisgarh, India. Women and Birth. 2018;31(2):e122–33. 10.1016/j.wombi.2017.07.003 28756932

[25] Korukcu O, Bulut O, Kukulu K. Psychometric Evaluation of the Wijma Delivery Expectancy/Experience Questionnaire Version B. Health Care Women Int. 2016;37(5):550–67. 10.1080/07399332.2014.943838 25119342

[26] König J. The German W-DEQ version B—Factor structure and prediction of posttraumatic stress symptoms six weeks and one year after childbirth. Health Care Women Int. 2019;40(5):581–96. 10.1080/07399332.2019.1583230 30901296

[27] Mortazavi F. Validity and reliability of the Farsi version of Wijma delivery expectancy questionnaire: an exploratory and confirmatory factor analysis. Electron Physician. 2017;9(6):4606–15. 10.19082/4606 28848637 PMC5557142

[28] Takegata M, Haruna M, Matsuzaki M, Shiraishi M, Murayama R, Okano T, et al. Translation and validation of the Japanese version of the Wijma Delivery Expectancy/Experience Questionnaire version A. Nurs Heal Sci. 2013;15(3):326–32. 10.1111/nhs.12036 23425355

[29] Ortega-Cejas CM, Roldán-Merino J, Lluch-Canut T, Castrillo-Pérez MI, Vicente-Hernandez MM, Jimenez-Barragan M, et al. Reliability and validity study of the Spanish adaptation of the “Wijma Delivery Expectancy/Experience Questionnaire" (W-DEQ-A). PLoS One. 2021;1–17. 10.1371/journal.pone.0248595 PMC7978360 33740006

[30] Tabachnick BG, Fidell LS. Multivariate analysis of variance and covariance. Using Multivar Stat. 2007;3:402–7.

[31] Streiner DL, Norman G, Cairney J. Health Measurement Scales:A Practical Guide to their Development and Use, 5th Edition. In: Oxford University Press. 2015.

[32] Wijma K, Wijma B, Zar M. Psychometric aspects of the W-DEQ; A new questionnaire for the measurement of fear of childbirth. J Psychosom Obstet Gynaecol. 1998;19(2):84–97. 10.3109/01674829809048501 9638601

[33] Frey BB. Standards for Educational and Psychological Testing. In: The SAGE Encyclopedia of Educational Research, Measurement, and Evaluation. 2018.

[34] Byrne BM. Structural Equation Modeling With EQS. Structural Equation Modeling With EQS. 2013.

[35] Browne M. W., and Cudeck R. Alternative ways of assessing model fit. In Bollen K. A. and Long J. S. (Eds.), Testing structural equation models. In: Newbury Park, CA: Sage. 1993.

[36] Kline RB. Principles and practice of structural equation modeling: Third Edition. Vol. 156, Structural Equation Modeling. 2011. 427 p.

[37] Brown TA. Confirmatory factor analysis for applied research. Second Edi. Confirmatory factor analysis for applied research. New York: The Guildford Press; 2015. 462 p.

[38] Byrne BM. Structural Equation Modeling With AMOS. Structural Equation Modeling With AMOS. 2016.

[39] Rial A, Varela J, Abalo J, Lévy JP. El análisis factorial confirmatorio. In: Lévy JP, Varela J, editors. Modelización con estructuras de covarianzas en ciencias sociales: temas esenciales, avanzados y aportaciones especiales. España: Gesbiblo S. L.; 2006. p. 119–54.

[40] Horn JL. A rationale and test for the number of factors in factor analysis. Psychometrika. 1965;30:179–85. 10.1007/BF02289447 14306381

[41] Ruscio J, Roche B. Determining the number of factors to retain in an exploratory factor analysis using comparison data of known factorial structure. Psychol Assess. 2012;24(2):282–92. 10.1037/a0025697 21966933

[42] Ferrando, Lorenzo-Seva U. Unrestricted item factor analysis and some relations with item response theory [Internet]. Department of Psychology, Universitat Rovira i Virgili, Tarragona; 2013. Available from: http://psico.fcep.urv.es/utilitats/factor.

[43] Ferrando PJ, Lorenzo-Seva U. Program FACTOR at 10: Origins, development and future directions. Psicothema. 2017;29:236–40. 10.7334/psicothema2016.304 28438248

[44] Lorenzo-Seva U, Ferrando PJ. Robust Promin: A method for diagonally weighted factor rotation. Lib Rev Peru Psicol. 2019;25(1):99–106.

[45] Cronbach LJ. Coefficient alpha and the internal structure of tests. Psychometrika. 1951;16(3):297–334.

[46] McDonald RP. Reliability Theory for Total Test Scores. In: Test Theory: A Unified Treatment. 2013. p. 62–75.

[47] Fenwick J, Gamble J, Nathan E, Bayes S, Hauck Y. Pre-and postpartum levels of childbirth fear and the relationship to birth outcomes in a cohort of Australian women. J Clin Nurs. 2009;18(5):667–77. 10.1111/j.1365-2702.2008.02568.x 19239535

[48] Wiklund I, Edman G, Ryding EL, Andolf E. Expectation and experiences of childbirth in primiparae with caesarean section. BJOG An Int J Obstet Gynaecol. 2008;115(3):324–31. 10.1111/j.1471-0528.2007.01564.x 18190368

[49] Abdi H, Williams LJ. Principal component analysis. Wiley Interdiscip Rev Comput Stat. 2010;433–59.

[50] Takegata M, Haruna M, Matsuzaki M, Shiraishi M, Okano T, Severinsson E. Psychometric Evaluation of the Japanese Wijma Delivery Expectancy/Experience Questionnaire Version B. Open J Nurs. 2017;7(1):15–27. 10.1111/nhs.12036 23425355

[51] Nunnally JC, Bernstein IH. The theory of measurement error. In: Psychometric Theory. 1994. p. 209–47.

[52] Waltz CF, Strickland OL, Lenz ER. Measurement in Nursing and Health Research. Vol. 22, Human Movement Science. New York, NY: Springer; 2010. 506 p.

[53] Kim H, Ku B, Kim JY, Park YJ, Park YB. Confirmatory and exploratory factor analysis for validating the phlegm pattern questionnaire for healthy subjects. Evidence-based Complement Altern Med. 2016;(2016):2696019. 10.1155/2016/2696019 27051447 PMC4804052

[54] Zou GY. Sample size formulas for estimating intraclass correlation coefficients with precision and assurance. Stat Med. 2012;31(29):3972–81. 10.1002/sim.5466 22764084

[55] Fleiss JL, Levin B, Paik MC. Statistical Methods for Rates and Proportions. Statistical Methods for Rates and Proportions. 2003.

[56] Cicchetti D V. Guidelines, Criteria, and Rules of Thumb for Evaluating Normed and Standardized Assessment Instruments in Psychology. Psychol Assess. 1994;6(4):284–90.

