# Supplementary material for: Validity and reliability of the Spanish version of the “Wijma Delivery Expectancy/Experience Questionnaire” (W-DEQ-B)
Source: PLoS One. 2021 Apr 26;16(4):e0249942. doi: 10.1371/journal.pone.0249942 (PMC8075224; doi:10.1371/journal.pone.0249942)
Supplement: S2 File — (DOC) [file pone.0249942.s002.doc]

**The Wijma Delivery Expectancy/Experience Questionnaire W-DEQ-B_Sp**

INSTRUCCIONES

Este cuestionario trata los sentimientos y pensamientos que pueden tener las mujeres después del parto.

Las respuestas a cada pregunta aparecen con una escala de 0 a 5. Los extremos de las respuestas (0 y 5 respectivamente) corresponden a los opuestos de un sentimiento o pensamiento.

Por favor, complete cada pregunta marcando con un círculo el número que más se acerca a **cómo piensa ahora que fue** su parto.

Por favor, responda a **cómo piensa que fue** su parto – no de la manera que hubieras deseado que fuera-.

1. **¿Cómo vivió el proceso de parto en general?**

| **1** | 0 | 1 | 2 | 3 | 4 | 5 |
| --- | --- | --- | --- | --- | --- | --- |
|  | Extremadamente fantástico |  |  |  |  | Nada fantástico |
| **2** | 0 | 1 | 2 | 3 | 4 | 5 |
|  | Extremadamente horrible |  |  |  |  | Nada horrible |

1. ¿**Cómo se sintió en general durante el proceso de parto?**

| **3** | 0 | 1 | 2 | 3 | 4 | 5 |
| --- | --- | --- | --- | --- | --- | --- |
|  | Extremadamente sola |  |  |  |  | Nada sola |
| **4** | 0 | 1 | 2 | 3 | 4 | 5 |
|  | Extremadamente fuerte |  |  |  |  | Nada fuerte |
| **5** | 0 | 1 | 2 | 3 | 4 | 5 |
|  | Extremadamente confiada |  |  |  |  | Nada confiada |
| **6** | 0 | 1 | 2 | 3 | 4 | 5 |
|  | Extremadamente asustada |  |  |  |  | Nada asustada |
| **7** | 0 | 1 | 2 | 3 | 4 | 5 |
|  | Extremadamente desatendida |  |  |  |  | Nada desatendida |
| **8** | 0 | 1 | 2 | 3 | 4 | 5 |
|  | Extremadamente débil |  |  |  |  | Nada débil |
|  |  |  |  |  |  |  |
| **9** | 0 | 1 | 2 | 3 | 4 | 5 |
|  | Extremadamente segura |  |  |  |  | Nada segura |
| **10** | 0 | 1 | 2 | 3 | 4 | 5 |
|  | Extremadamente independiente |  |  |  |  | Nada independiente |
| **11** | 0 | 1 | 2 | 3 | 4 | 5 |
|  | Extremadamente desolada |  |  |  |  | Nada desolada |
| **12** | 0 | 1 | 2 | 3 | 4 | 5 |
|  | Extremadamente tensa |  |  |  |  | Nada tensa |
| **13** | 0 | 1 | 2 | 3 | 4 | 5 |
|  | Extremadamente contenta |  |  |  |  | Nada contenta |
| **14** | 0 | 1 | 2 | 3 | 4 | 5 |
|  | Extremadamente orgullosa |  |  |  |  | Nada orgullosa |
| **15** | 0 | 1 | 2 | 3 | 4 | 5 |
|  | Extremadamente abandonada |  |  |  |  | Nada abandonada |
| **16** | 0 | 1 | 2 | 3 | 4 | 5 |
|  | Totalmente íntegra |  |  |  |  | Nada íntegra |
| **17** | 0 | 1 | 2 | 3 | 4 | 5 |
|  | Extremadamente relajada |  |  |  |  | Nada relajada |
| **18** | 0 | 1 | 2 | 3 | 4 | 5 |
|  | Extremadamente feliz |  |  |  |  | Nada feliz |

1. **¿Qué sentiste durante el proceso de parto?**

| **19** | 0 | 1 | 2 | 3 | 4 | 5 |
| --- | --- | --- | --- | --- | --- | --- |
|  | Pánico extremo |  |  |  |  | Ningún pánico |
| **20** | 0 | 1 | 2 | 3 | 4 | 5 |
|  | Desesperanza extrema |  |  |  |  | Ninguna desesperanza |
| **21** | 0 | 1 | 2 | 3 | 4 | 5 |
|  | Deseo extremo por el bebé |  |  |  |  | Ningún deseo por el bebé |
| **22** | 0 | 1 | 2 | 3 | 4 | 5 |
|  | Autoconfianza extrema |  |  |  |  | Ninguna autoconfianza |
| **23** | 0 | 1 | 2 | 3 | 4 | 5 |
|  | Confianza extrema |  |  |  |  | Ninguna confianza |
| **24** | 0 | 1 | 2 | 3 | 4 | 5 |
|  | Dolor extremo |  |  |  |  | Ningún dolor |

1. **¿Qué paso cuando el trabajo de parto fue más intenso?**

| **25** | 0 | 1 | 2 | 3 | 4 | 5 |
| --- | --- | --- | --- | --- | --- | --- |
|  | Me comporté extremadamente mal |  |  |  |  | No me comporté mal en absoluto |
| **26** | 0 | 1 | 2 | 3 | 4 | 5 |
|  | Permití que mi cuerpo tomara el control total |  |  |  |  | No permití que mi cuerpo tomara el control total |
| **27** | 0 | 1 | 2 | 3 | 4 | 5 |
|  | Perdí el control total de mí misma |  |  |  |  | No perdí el control de mí misma en absoluto |

1. **¿Cómo fue el momento de la salida del bebé?**

| **28** | 0 | 1 | 2 | 3 | 4 | 5 |
| --- | --- | --- | --- | --- | --- | --- |
|  | Extremadamente agradable |  |  |  |  | Nada agradable |
| **29** | 0 | 1 | 2 | 3 | 4 | 5 |
|  | Extremadamente natural |  |  |  |  | Nada natural |
| **30** | 0 | 1 | 2 | 3 | 4 | 5 |
|  | Totalmente como debía ser |  |  |  |  | En absoluto como debía ser |
| **31** | 0 | 1 | 2 | 3 | 4 | 5 |
|  | Extremadamente peligroso |  |  |  |  | Nada peligroso |

1. **¿Tuvo durante el trabajo de parto y nacimiento fantasías como, por ejemplo…**

**…Fantasías de que su bebé podría morir durante el trabajo de parto y nacimiento?**

| **32** | 0 | 1 | 2 | 3 | 4 | 5 |
| --- | --- | --- | --- | --- | --- | --- |
|  | Nunca |  |  |  |  | Muy a menudo |

**…Fantasías de que su bebé podría lesionarse durante el trabajo de parto y nacimiento?**

| **33** | 0 | 1 | 2 | 3 | 4 | 5 |
| --- | --- | --- | --- | --- | --- | --- |
|  | Nunca |  |  |  |  | Muy a menudo |

¿Por favor, podría comprobar que no ha olvidado responder ninguna pregunta?
